# Psychiatric emergency department visits during the coronavirus disease-2019 pandemic

**DOI:** 10.3389/fpsyt.2023.1236584

**Published:** 2023-08-28

**Authors:** HaiMing Sun, HaiChun Liu, ChunYan Ma, Zheng Chen, YanYan Wei, XiaoChen Tang, LiHua Xu, YeGang Hu, YuOu Xie, Tao Chen, Zheng Lu, JiJun Wang, TianHong Zhang

**Affiliations:** ^1^Shanghai Key Laboratory of Psychotic Disorders, Shanghai Mental Health Center, Shanghai Jiao Tong University School of Medicine, Shanghai Engineering Research Center of Intelligent Psychological Evaluation and Intervention, Shanghai, China; ^2^Department of Automation, Shanghai Jiao Tong University, Shanghai, China; ^3^The First Clinical Medical College of Nanjing Medical University, Nanjing, China; ^4^Big Data Research Lab, University of Waterloo, Waterloo, ON, Canada; ^5^Labor and Worklife Program, Harvard University, Cambridge, MA, United States; ^6^Department of Psychiatry, Tongji Hospital, Tongji University School of Medicine, Shanghai, China; ^7^Center for Excellence in Brain Science and Intelligence Technology (CEBSIT), Chinese Academy of Science, Shanghai, China; ^8^Institute of Psychology and Behavioral Science, Shanghai Jiao Tong University, Shanghai, China

**Keywords:** psychiatric hospital, stress, anxiety disorder, sleep disorder, emergency psychiatry

## Abstract

**Background:**

Previous research has demonstrated the negative impact of the coronavirus disease-2019 (COVID-19) pandemic on mental health.

**Aims:**

To examine changes in the Chinese psychiatric emergency department (PED) visits for mental health crises that occurred during the pandemic.

**Methods:**

Before and during the COVID-19 pandemic, PED visit counts from the largest psychiatric hospital in China between 2018 and 2020 were investigated. Electronic medical records of 2020 PED visits were extracted during the COVID-19 pandemic period and compared for the same period of 2018 and 2019.

**Results:**

Overall, PED visits per year increased from 1,767 in 2018 to 2210 (an increase of 25.1%) in 2019 and 2,648 (an increase of 49.9%) in 2020. Compared with 2 years before the epidemic, during the COVID-19 pandemic, the proportion of PED visits among patients with stress disorders, sleep disorders, and anxiety disorders increased significantly. In terms of the distribution of demographic characteristics, age shows a younger trend, while the gender difference is not significant.

**Conclusion:**

These findings suggest that PED care-seeking increases during the COVID-19 pandemic, highlighting the need to integrate mental health services for patients with stress, sleep, anxiety, and obsessive-compulsive disorders during public health crises.

## Background

The coronavirus disease-2019 (COVID-19) pandemic has characterized today's society and interpersonal relationships as guarded and distant. Since the evolution of COVID-19 remains unpredictable, the impact of the pandemic on mental health may persist for a long time (1). Previous surveys of the general public (2–4) and patients with psychiatric disorders (5–7) have shown increased symptoms of anxiety, stress, and depressive mood during the COVID-19 pandemic. Epidemic prevention and administrative restrictions may cause home confinement, disruption of daily routines, and physical distancing, further limiting scarce access to mental health services, which could exacerbate mental health conditions (8).

Although self-reported psychiatric symptoms have increased significantly during the epidemic in a large number of studies (9–11), this finding does not directly support the increase in mental disorders. One of the most direct evidence is the change in the number of psychiatric emergency patients because emergency services are for patients with severe, crisis, or acute mental disorders, which have a clear relationship with the episode or deterioration of mental disorders. Unfortunately, the research results are inconsistent in different regions due to control policies. The number of visits to the psychiatric emergency department (PED) could be decreased by a “stay-at-home” order (12) or a fear of COVID-19 infection (13, 14), varied by region (15) and increased by the deconfinement period (16).

The impact of the COVID-19 pandemic has been found to be more severe on adolescents compared to adults. Adolescence is a crucial developmental stage characterized by significant physical, cognitive, and social changes. The disruptions caused by the pandemic, such as school closures, social isolation, and limited access to support systems, have disproportionately affected the mental health and wellbeing of adolescents. Adolescents rely heavily on social interactions and peer relationships for their emotional and social development. The restrictions imposed during the pandemic, including physical distancing measures and remote learning, have resulted in decreased opportunities for in-person socialization and increased feelings of loneliness and isolation among adolescents. These factors contribute to higher levels of stress, anxiety, and depressive symptoms in this population. Additionally, adolescents may experience heightened emotional reactivity and have limited coping mechanisms to deal with the stressors brought on by the pandemic. They may face difficulties in expressing their emotions and seeking appropriate support, further exacerbating their mental health struggles. Recognizing the unique vulnerabilities of adolescents during this challenging time is essential for developing targeted interventions and support systems. Providing accessible mental health services, promoting social connectivity through virtual platforms, and incorporating mental health education into remote learning curricula are crucial steps in mitigating the negative impact of the pandemic on adolescents' mental wellbeing (17, 18).

Government reactions to the pandemic, economic considerations, and epidemic prevention measures and implementation varied greatly between countries. China is one of the few countries still adhering to the principle of a “dynamic zero-COVID policy” (19) to treat COVID-19. To achieve the aim of “dynamic zeroing,” the Chinese government issued the strictest epidemic prevention measures, such as stay-at-home orders, home-based distance learning or working models, and shutting down non-essential community services, which has resulted in physical distancing and may have long-term negative consequences for mental health (20). These characteristics of China make the data on the use of PED incomparable with those of Western countries. However, no studies have evaluated the changes in PED visits during the COVID-19 pandemic in China. Although surveys on the impact of COVID-19 on PED visits are informative in other countries (12, 21–23), there is a particular need to evaluate whether changes in frequency and/or patient demographics have occurred among Chinese psychiatric patients visiting PED. To test this hypothesis, we examined the number of PED visits in different diagnostic categories in Shanghai, China (obtained from the largest psychiatric hospital in China) from 2018 to 2020.

## Methods

### Data collection

This cross-sectional study was a retrospective analysis of the number of patients observed in PED at the Shanghai Mental Health Center (SMHC). The administrative dataset for the years 2018–2020 was used in this study. SMHC is the largest outpatient mental health clinic in China, offering medication management and psychotherapy in Shanghai, with over 1 million outpatients per year. The patients come from different parts of the country. The emergency department at the SMHC is the largest PED and provides 24-h service all year round. It is the only referral hospital in Shanghai that serves all psychiatric emergency patients. The PED visits are attended by a team of 10 experienced psychiatric doctors who specialize in handling mental health emergencies. Each of these doctors has a wealth of expertise in the field of psychiatry, with more than 10 years of professional experience. Furthermore, they had undergone rotations in internal medicine emergency departments for at least 6 months, providing them with additional knowledge and skills in managing acute medical conditions.

The study population was identified as all patients who visited the PED at the SMHC in 2018, 2019, and 2020. The dates of PED visits were extracted based on the number of patients, diagnosis, age, and sex. All data were de-identified. The Research Ethics Committees at the SMHC approved this study. This study should be considered public health surveillance rather than research involving human subjects; therefore, informed consent was waived for these secondary data analyses. The duration of COVID-19 influence in Shanghai was settled from 0:00 h on 1 January 2020 to 24:00 h on 31 December 2020, since during the COVID-19 epidemic, and 2018–2019 years were treated as controls before the epidemic.

### Diagnostic categories

Individual diagnostic information was extracted from the SMHC diagnosis and treatment system database and recorded in the administrative dataset, which was mainly based on codes from the International Classification of Diseases version 10 (ICD-10). The diagnostic categories used in this analysis were as follows: (1) AD: Alzheimer's disease and other dementias; (2) ID: intellectual disability; (3) TIC: tic disorder; (4) epilepsy; (5) delirium; (6) SARD: substance abuse and related disorders; (7) PDRD: physical diseases and related disorders; and (8) State: a diagnosis was given at visits in which the examined patient was in a state of excitement and agitation, silence, chaos, brought for psychiatric evaluation for safety evaluation. The excitement state accounts for the majority: (9) psychosis; (10) Mood-D: mood disorders; (11) Stress-D: stress-related disorders; (12) Sleep-D: sleep disorders; (13) Dissociative-D: dissociative disorders; (14) Anxiety-D: anxiety disorders; (15) OCD: obsessive-compulsive disorder; and (16) TBD: to be determined, that is, those whose diagnosis was unclear.

### Data analysis

The number of patients in the PED visit counts was presented and compared monthly and stratified by age, sex, and diagnostic categories. Statistical analysis was performed to compare the monthly numbers of PED visits in 2018–2020. The ratio of diagnostic categories is presented as frequencies and percentages of the total sample for those 3 years. All datasets were transferred to Excel spreadsheets (Excel; Microsoft Corporation, Redmond, WA, USA), and pie and bar graphs were generated using the software. We used means and standard deviations for continuous variables and frequencies and percentages for categorical variables. The variables were compared between groups using a one-way ANOVA (for continuous variables) or a chi-squared test (for categorical variables). Statistical significance was set at a *p*-value of < 0.05.

## Results

**Figure 1:**
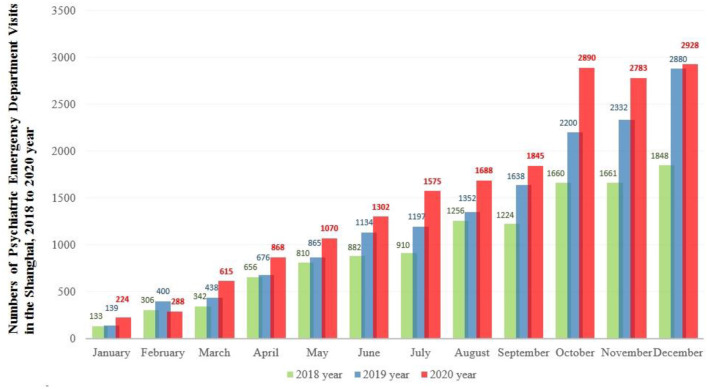
The number of monthly psychiatric emergency department visits across 2018, 2019, and 2020.

The number of PED visits per year increased from 1,767 in 2018 to 2,210 (an increase of 25.1%) in 2019 and 2,648 (an increase of 49.9%) in 2020. The descriptive profile of the monthly PED visits over 3 years is presented in Figure 1.

**Figure 2:**
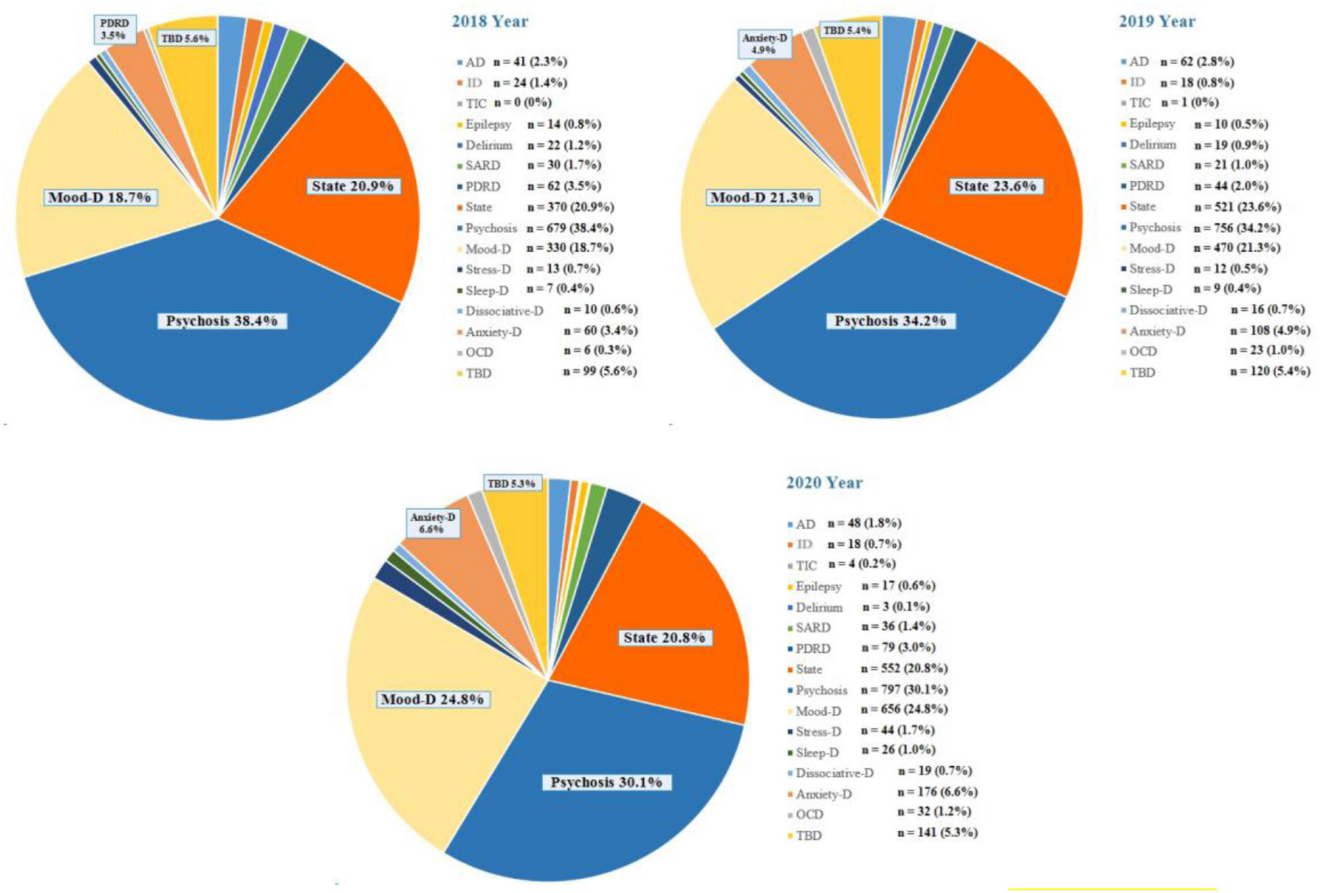
The number of psychiatric emergency department visits stratified by various diagnostic categories, 2018–2020. Diagnostic categories: AD, Alzheimer's disease and other dementias; ID, intellectual disability; TIC, Tic disorder; Epilepsy; Delirium; SARD, Substance abuse and related disorders; PDRD, Physical diseases and related disorders; State, Excitement state account for the majority; Psychosis; Mood-D, Mood disorders; Stress-D, Stress-related disorders; Sleep-D, Sleep disorders; Dissociative-D, Dissociative Disorders; Anxiety-D, Anxiety Disorders; OCD, Obsessive-compulsive disorder; TBD, To be determined.

As shown in Figure 2, the diagnostic categories of psychosis, mood disorders, and state were commonly used in patients visiting the PED (the total proportion was >75%). Although the top three diagnostic categories are relatively stable, the proportion of psychosis in these 3 years declined, while the proportion of depression rose. In particular, the proportion of anxiety disorders did not make into the top five in 2018 but gradually increased from 3.4% to 6.6% in 2019 (top-5) and 2020 (top-4).

**Figure 3:**
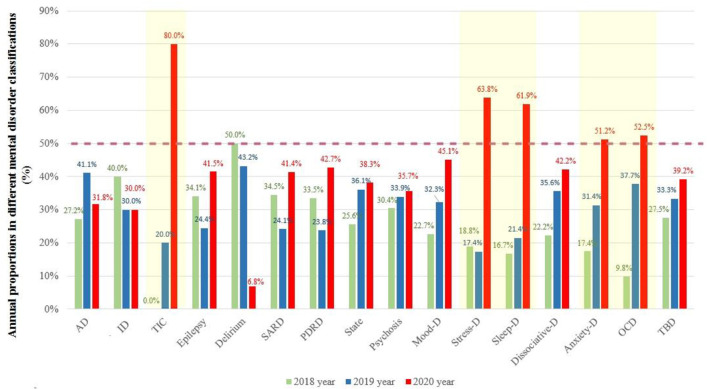
The annual proportion of patients who visit PED in each diagnostic category. Diagnostic categories: AD, Alzheimer's disease and other dementias; ID, intellectual disability; TIC, Tic disorder; Epilepsy; Delirium; SARD, Substance abuse and related disorders; PDRD, Physical diseases and related disorders; State, Excitement state account for the majority; Psychosis; Mood-D, Mood disorders; Stress-D, Stress-related disorders; Sleep-D, Sleep disorders; Dissociative-D, Dissociative disorders; Anxiety-D, Anxiety disorders; OCD, Obsessive-compulsive disorder; TBD, To be determined.

To further compare the changes in the distribution of diagnostic categories between 2020 and 2018–2019. Figure 3 shows the annual proportion of patients who visit PED in each diagnostic category. In 2020, the volume of PED visits in patients with Tic disorder, Stress-D, Sleep-D, Anxiety-D, and OCD accounted for more than half of the 3-year total.

Table 1 describes age differences from 2018 to 2020, and comparisons of the mean age are applied in each diagnostic category. Patients with anxiety disorders in 2020 PED visits were younger, while patients with epilepsy or OCD were older than those in the years 2018–2019.

**Table 1:** Age profile of patients using psychiatric emergency in 2018–2020 years in diagnostic categories.

**Diagnostic categories**	**2018**	**2019**	**2020**	**Comparisons**	
				***F***	***p***
AD	71.4 (12.4)	75.0 (11.5)	74.3 (13.1)	1.091	0.338
ID	46.5 (21.0)	43.1 (20.7)	41.4 (15.8)	0.38	0.685
TIC	-	17.0 (-)	20.6 (4.9)	0.438	0.555
Epilepsy	41.5 (16.9)	53.5 (13.8)	65.4 (27.6)	4.737	**0.015**
Delirium	72.3 (14.0)	72.8 (15.1)	79.8 (18.6)	0.353	0.705
SARD	45.6 (12.7)	48.6 (13.7)	43.1 (11.8)	1.276	0.284
PDRD	58.7 (21.8)	58.3 (17.4)	62.4 (20.6)	0.826	0.439
State	39.8 (17.8)	39.7 (17.8)	39.6 (16.8)	0.019	0.981
Psychosis	39.3 (13.3)	40.1 (13.4)	40.4 (14.2)	1.161	0.313
Mood-D	40.1 (17.4)	38.0 (16.8)	39.0 (17.2)	1.371	0.254
Stress-D	45.0 (16.2)	36.3 (14.4)	42.7 (16.0)	1.061	0.352
Sleep-D	45.6 (19.9)	34.3 (9.2)	44.9 (18.4)	1.383	0.263
Dissociative-D	54.9 (11.8)	51.5 (15.2)	43.2 (12.8)	2.994	0.061
Anxiety-D	46.1 (15.1)	47.0 (17.5)	39.4 (16.2)	8.51	**< 0.001**
OCD	23.2 (7.3)	36.4 (13.9)	41.2 (15.2)	4.24	**0.019**
TBD	36.2 (16.6)	39.0 (16.3)	39.0 (17.0)	1.067	0.345

Diagnostic categories: AD, Alzheimer's disease and other dementias; ID, intellectual disability; TIC, Tic disorder; Epilepsy; Delirium; SARD, Substance abuse and related disorders; PDRD, Physical diseases and related disorders; State, Excitement state account for the majority; Psychosis; Mood-D, Mood disorders; Stress-D, Stress-related disorders; Sleep-D, Sleep disorders; Dissociative-D, Dissociative Disorders; Anxiety-D, Anxiety Disorders; OCD, Obsessive-compulsive disorder; TBD, To be determined. F for the one-way ANOVA. Bold in significant.

Patients who visited PED were further stratified by sex and diagnostic categories and compared over the years (Table 2). Overall, the sex ratio was generally similar in these 3 years, with the only exception being the increase in the proportion of female patients with psychosis in PED visits from 2018 to 2020.

**Table 2:** Sex profile of patients using psychiatric emergency in 2018–2020 years in diagnostic categories (Female, %).

**Diagnostic categories**	**2018**	**2019**	**2020**	**Comparisons**	
				*χ^2^*	***p***
AD	13 (31.7)	26 (41.9)	23 (47.9)	2.434	0.296
ID	7 (29.2)	8 (44.4)	7 (38.9)	1.089	0.58
TIC	-	1 (100)	4 (100)	-	-
Epilepsy	2 (14.3)	2 (20.0)	8 (47.1)	4.532	0.104
Delirium	10 (45.5)	6 (31.6)	1 (33.3)	0.866	0.649
SARD	4 (13.3)	3 (14.3)	9 (25.0)	1.795	0.408
PDRD	19 (30.6)	18 (40.9)	28 (35.4)	1.195	0.55
State	202 (54.6)	302 (58.0)	329 (59.6)	2.295	0.317
Psychosis	351 (51.7)	420 (55.6)	477 (59.8)	9.953	**0.007**
Mood-D	208 (63.0)	301 (64.0)	373 (62.2)	0.398	0.82
Stress-D	9 (69.2)	7 (58.3)	31 (70.5)	0.647	0.724
Sleep-D	5 (71.4)	8 (88.9)	19 (73.1)	1.026	0.599
Dissociative-D	6 (60.0)	14 (87.5)	16 (84.2)	3.273	0.195
Anxiety-D	33 (55.0)	65 (60.2)	115 (65.3)	2.23	0.328
OCD	2 (33.3)	8 (34.8)	13 (40.6)	0.249	0.883
TBD	49 (49.5)	65 (54.2)	94 (47.7)	1.255	0.534

Diagnostic categories: AD, Alzheimer's disease and other dementias; ID, intellectual disability; TIC, Tic disorder; Epilepsy; Delirium; SARD, Substance abuse and related disorders; PDRD, Physical diseases and related disorders; State, Excitement state account for the majority; Psychosis; Mood-D, Mood disorders; Stress-D, Stress-related disorders; Sleep-D, Sleep disorders; Dissociative-D, Dissociative Disorders; Anxiety-D, Anxiety Disorders; OCD, Obsessive-compulsive disorder; TBD, To be determined. χ^2^ for kappa test. Bold in significant.

## Discussion

Psychiatric emergencies are at the forefront of the treatment of a mental illness crisis. To our knowledge, this is the first study to consider the impact of COVID-19 on the use of PED in a psychiatric hospital in China. This cross-sectional study provides hospital encounter data showing a potential association between the pandemic and an increase in the total number of PED visits, specifically in patients with stress-D, sleep-D, anxiety-D, and OCD. Consistently, studies have documented self-reported stressful experiences (24), sleep problems (25), anxiety (26), and obsessive-compulsive symptoms (27) in the Chinese population during the pandemic.

Along with protracted infection containment strategies in China, the number of PED visits has consistently increased compared to the pre-pandemic period. The only exception was February 2020, when near the beginning of the pandemic and strict implementation of the quarantine strategy, the number of PED visits decreased compared to the same period in the previous 2 years (Figure 1). With the clarification of the “dynamic zero-COVID policy,” the mental health service response to COVID-19 should not only provide information on how to cope with epidemic phobia but also should aim to increase the capacity to treat mental disorder crises. In particular, visits to PED in 2020 were likely to be of sufficient severity, and seeking help in a PED was a necessary risk during the pandemic, despite the stay-at-home orders that advised people to avoid public spaces.

An unexpected finding in this study was that the number of PED visits from October to December increased over the past 3 years compared to previous months. While the information provided does not explicitly mention the specific factors contributing to the increased number of PED visits from October to December, some potential reasons could be inferred based on general trends in mental health. First, seasonal changes, particularly the transition from autumn to winter, can lead to an increase in individuals experiencing seasonal affective disorder (SAD) (28). SAD is a type of depression that typically occurs during the fall and winter months when there is less sunlight, and it can exacerbate existing mental health conditions, leading to more people seeking mental health support during this time. Second, in many countries, October to December marks the end of the year in terms of academic semesters and fiscal quarters. Students may experience increased stress due to final exams and assignments, while employees may feel pressure to meet year-end targets (29, 30). Such heightened stress levels could contribute to the rise in mental health emergencies during this period. Finally, in certain regions, winter weather conditions can have a negative impact on mental wellbeing. Cold, dark, and gloomy weather can affect people's moods and exacerbate symptoms of depression and anxiety (31). The information provided in the original study does not specify these factors, so further research would be needed to explore the specific reasons behind the observed pattern in PED visits during this period.

The findings of this study revealed that there was no significant gender difference in PED visits during the COVID-19 pandemic. This result contrasts with some previous studies (32, 33) that have reported a greater impact on women's mental health during public health crises. The discrepancy between our findings and previous research could be attributed to various factors. First, cultural and contextual differences may play a role in shaping help-seeking behaviors and in the manifestation of mental health symptoms. Societal norms, gender roles, and expectations can influence the expression and recognition of mental health issues among different populations. Second, the study focused on PED visits, which represent individuals seeking emergency psychiatric care. It is possible that the observed gender differences in previous studies were related to differences in help-seeking patterns rather than the actual prevalence of mental health conditions. Women may be more inclined to seek help and utilize mental health services, leading to higher reported rates of mental healthcare utilization. Third, it is essential to consider the specific sample characteristics and study design when interpreting the findings. Further research is warranted to explore the underlying factors contributing to the observed lack of significant gender difference in PED visits during the pandemic. Qualitative studies or surveys that capture individuals' experiences and perceptions of mental health during the crisis may provide valuable insights into the nuances of gender differences in help-seeking behaviors and mental health outcomes.

Stress, sleep, and anxiety disorders exhibited greater increases in overall visits in 2020 than in 2018 and 2019. This increase indicates that the number of patients in 2020 was more than half of the total number in 3 years, which is an especially compelling evidence, suggesting an increase in the burden of those disorders during the pandemic. Tsur et al. (34) assessed cross-national acute stress symptoms following the initial COVID-19 outbreak in China, Israel, and Switzerland and revealed that, despite some variations, the overall clinical picture of pandemic-related stress symptoms is universal. Furthermore, consistent with our finding of increased PED visits in patients with OCD, Ji et al. (27) reported that the prevalence of possible OCD in the early stage of the COVID-19 pandemic was significantly higher than that in the middle-to-late stages, suggesting that the intensity of fear of COVID-19 played a role in OCD and may be associated with fear, anxiety, and pandemic-induced quarantine.

In a further secondary analysis of age, an important finding in this study was that the number of PED visits by patients with anxiety disorders was lower in 2020 than in the pre-pandemic years. The youth, a vulnerable population (35), have been attending their school curriculum online during the pandemic in China. Younger people who have never experienced an epidemic in their lifetime may be less mature and perhaps incapable of facing this lifestyle transformation and the threat of being infected, which in turn could lead to serious anxiety disorders (10). Moreover, this study found that the proportion of female patients with psychosis during PED visits increased from 2018 to 2020. The narrative review by Almeida et al. (36) summarized that the pandemic predisposes female patients to vulnerabilities and adverse impacts on mental health more than male patients, especially those who are pregnant, postpartum, miscarrying, or experiencing intimate partner violence.

The findings of this study provide valuable insights that can inform the development of specific strategies for PED during future significant public health events. Understanding the patterns and changes in PED visits during the COVID-19 pandemic can guide policymakers, healthcare providers, and emergency management teams in preparing for and responding to similar crises in future. The significant increase in PED visits observed during the pandemic highlights the need for adequate allocation of resources, including staffing, facilities, and supplies, during public health emergencies. Policymakers and hospital administrators can use the findings to anticipate and prepare for the increased demand for PED services, ensuring that sufficient resources are available to meet the surge in mental healthcare needs. The study identified a higher proportion of PED visits among patients with stress disorders, sleep disorders, anxiety disorders, and obsessive-compulsive disorders during the pandemic. This information can guide the development of targeted mental health services within the PED settings, such as specialized assessment and treatment protocols for these specific conditions. Integration of evidence-based interventions and support systems tailored to address stress, sleep, anxiety, and obsessive-compulsive disorders can enhance the effectiveness of emergency mental healthcare.

The study noted a younger trend in the age distribution of PED visitors during the pandemic. This finding emphasizes the importance of age-specific interventions and support for adolescents and young adults during public health emergencies. Healthcare providers can develop age-appropriate strategies to address the unique mental health challenges faced by this population, including targeted outreach, telehealth services, and educational initiatives focused on building resilience and coping skills (30). The increased PED visits during the pandemic highlight the need for integration and collaboration between psychiatric and medical emergency departments. Collaborative care models can facilitate seamless coordination and communication between different specialties, ensuring comprehensive care for patients with both mental health and medical needs during public health crises. The findings of this study highlight the importance of mental health preparedness in emergency response planning. Policymakers and healthcare organizations should prioritize mental health as an integral component of emergency preparedness, including the development of protocols, training programs, and guidelines for PED staff to effectively manage mental health emergencies during public health events. By incorporating these strategies, healthcare systems can better respond to the mental health needs of individuals during significant public health emergencies. The insights gained from this study can serve as a foundation for evidence-based decision-making and the development of proactive strategies to address the mental health burden during future crises (37).

## Limitations

Several limitations of this study should be considered when interpreting its findings. First and most importantly, this study had a single-site cross-sectional design. Consequently, the data are not nationally representative and the results may not be generalizable. However, the trends elucidated in Shanghai are likely to be generalizable to the entire country, considering that SMHC is the largest psychiatric hospital, taking over patients nationwide, and is highly representative of the overall demographics of China. Second, diagnostic categories may be transitional diagnoses when the first visit is recorded. The diagnostic category should not be interpreted as an exact diagnosis, because it may change over time. Third, some confounding factors, such as different emergency doctors and patients' exposure to the epidemic, may bias the prevalence of diagnostic categories in patients visiting PED. Finally, one notable limitation of this study is the absence of a structured interview to assess the severity of mental illness among PED visitors. While the study examined the changes in PED visit counts and the distribution of mental health conditions, it did not employ standardized measures or scales to quantify the severity of the identified conditions.

## Future research

Several areas warrant further investigation to enhance our understanding of the mental health impact and inform strategies for future emergencies. First, conducting longitudinal studies to examine the long-term effects of public health emergencies on PED utilization and mental health outcomes would provide a more comprehensive understanding of the trajectory of mental health conditions. Second, comparing the findings of this study with data from different geographic regions and cultural contexts would allow for a broader understanding of the global impact of public health emergencies on PED visits. Third, future research could explore the effectiveness, accessibility, and acceptability of remote mental health services in emergency settings. Fourth, studying the mental health impact on marginalized groups, including racial and ethnic minorities, low-income individuals, and individuals with pre-existing mental health conditions, would allow for targeted interventions and equitable mental health support. Fifth, comparing PED visit patterns, mental health conditions, and demographic characteristics before and after the pandemic would elucidate the unique impact of COVID-19 on mental health crises.

## Conclusion

Despite barriers against PED visits, such as fear of attending a hospital due to the risk of being infected and mandatory quarantine, the number of PED visits increased during the pandemic in Shanghai, China, particularly for patients with stress, sleep, anxiety, and obsessive-compulsive disorders. As reported in past research on public health crises (38, 39), we can assume a much greater need for psychiatric emergency interventions for the long-term consequences of a pandemic. Further observations should be made regarding the meaning of the increasing demands so that the needs are recognized and the system is ready.

## Data availability statement

The raw data supporting the conclusions of this article will be made available by the authors, without undue reservation.

## Ethics statement

The studies involving humans were approved by the Ethics Committee at Shanghai Mental Health Center. The studies were conducted in accordance with the local legislation and institutional requirements. The participants provided their written informed consent to participate in this study.

## Author contributions

TZ, HL, HS, CM, ZL, ZC, and JW: conceptualized the study, wrote the first draft of the manuscript, and conducted the statistical analyses. LX, YW, XT, and ZC: collected and organized the primary data. YH, HL, TC, and YX: managed the literature searches, statistical analyses, and edited the manuscript. JW and TZ designed the study and provided supervision in the implementation of the study. All authors have approved the final manuscript.
